# *DDX41* mutations establish a separate entity in acute myeloid leukemia with distinct survival outcomes

**DOI:** 10.1002/hem3.70483

**Published:** 2026-09-14

**Authors:** Carolin Seeling, Maral Saadati, Daniela Späth, Michael Heuser, Felicitas Thol, Arnold Ganser, Elli Papaemmanuil, Lars Bullinger, Axel Benner, Hartmut Döhner, Konstanze Döhner

**Affiliations:** ^1^ Department of Internal Medicine III University Hospital of Ulm Ulm Germany; ^2^ Division of Biostatistics German Cancer Research Center (DKFZ) Heidelberg Germany; ^3^ Department of Internal Medicine IV University Hospital Halle (Saale), Martin Luther University Halle‐Wittenberg Halle (Saale) Germany; ^4^ Department of Hematology, Hemostasis, Oncology and Stem Cell Transplantation Hannover Medical School Hannover Germany; ^5^ Department of Computational Oncology, Epidemiology and Biostatistics Memorial Sloan Kettering Cancer Center New York New York United States of America; ^6^ Department of Hematology, Oncology and Cancer Immunology Charité‐Universitätsmedizin Berlin, Corporate Member of Freie Universität Berlin, Humboldt‐Universität zu Berlin Berlin Germany; ^7^ German Cancer Consortium (DKTK) Partner Site Berlin Germany; ^8^ National Center for Tumor Diseases (NCT) Partner Site Berlin Germany

Recurrent germline variants in *DDX41* represent the most common inherited predisposition to adult myeloid neoplasms, occurring in approximately 3%–5% of patients with acute myeloid leukemia (AML) or myelodysplastic syndrome (MDS).^1^, ^2^

Clinically, patients with *DDX41*‐mutated AML are typically older at diagnosis, predominantly male, and present with lower white blood cell (WBC) counts.^3^, ^4^, ^5^, ^6^, ^7^, ^8^

Unlike other molecular subgroups of AML, *DDX41*‐mutated AML rarely harbors class‐defining genetic alterations such as *NPM1* mutations, biallelic *CEBPA* mutations, or core‐binding factor rearrangements and is instead characterized by few cooperating mutations and predominantly normal karyotypes.^3^, ^4^, ^5^, ^9^

These distinctive clinical and biological features have led to the recognition of *DDX41*‐associated myeloid neoplasms as a distinct disease entity. However, whether *DDX41*‐mutated AML constitutes a genetically distinct subgroup within the broader AML landscape remains incompletely defined. We therefore comprehensively characterized the molecular landscape and clinical outcome of *DDX41*‐mutated AML in two independent clinical trial cohorts and applied unsupervised clustering algorithms to determine whether these cases represent a distinct genomic entity.

The primary cohort comprised 516 younger patients (median age 53.5 years) receiving intensive therapy and included predefined AML subgroups selected on the basis of their known molecular phenotype, in which *DDX41* mutations were expected to be enriched.^10^ The extension cohort comprised 604 older patients (median age 77 years) receiving non‐intensive therapy without molecular preselection.^11^ Targeted sequencing data were integrated with existing molecular datasets and analyzed using Uniform Manifold Approximation and Projection (UMAP) followed by Density‐Based Spatial Clustering of Applications with Noise (DBSCAN). Overall survival (OS), event‐free survival (EFS), and relapse‐free survival (RFS) were analyzed using Kaplan–Meier estimates and multivariable Cox regression. Detailed methods are provided in the Supplementary Appendix.

Among the 516 intensively treated patients, 18 (3.5%) harbored pathogenic *DDX41* mutations. Patients with *DDX41*‐mutated AML were older at diagnosis than those with *DDX41*‐wildtype AML (median age, 58.5 vs. 53.0 years), predominantly male (77.8% vs. 59.0%), and presented with lower white blood cell counts (median, 2.1 × 10^9^/L vs. 9.2 × 10^9^/L; Table S1).

Pathogenic *DDX41* mutations were unevenly distributed across predefined molecular classes and occurred most frequently in the *No_drivers* subgroup (20%; 9/45), compared with 3% (4/128) in *No_class*, 2% (4/203) in *chromatin‐spliceosome*, and 1% (1/140) in *TP53‐aneuploidy* (P < 0.001). After adjustment for multiple testing for pairwise comparisons, the frequency in *No_drivers* remained significantly higher than in all other subgroups (Table S2), supporting the notion that *DDX41* mutations preferentially occur in the absence of other AML driver lesions.

The mutational spectrum of *DDX41* closely mirrored previous reports and was characterized by frequent biallelic alterations consisting of germline and somatic pathogenic *DDX41* variants (Figures S1 and S2; Table S10). The most frequent germline alteration was p.(Met1Ile), whereas p.(Arg525His) represented the predominant somatic hotspot mutation. Co‐occurring mutations were infrequent (median, 0.5; range, 0–3) and mainly involved *ASXL1* (16.7%), *PHF6* (16.7%), and *SRSF2* (11.1%). A normal karyotype was observed in 55.6% of cases. Overall, these findings are consistent with the characteristic low mutational complexity of *DDX41*‐mutated AML.

To explore the genomic structure of the cohort, mutation profiles were projected into two‐dimensional space using UMAP. Cases with pathogenic *DDX41* variants formed a tightly clustered group, indicating that *DDX41*‐mutated AML represents a distinct molecular entity (Figure 1A). Two cases mapped to clusters dominated by *TP53*‐mutated complex‐karyotype AML or *FLT3*‐ITD‐mutated AML, suggesting that these co‐occurring alterations dictated the dominant molecular phenotype. Notably, these cases lacked the canonical biallelic *DDX41* configuration (see Table S12 for additional data). Figure S3 provides UMAP plots for all analyzed genes.

**Figure 1:**
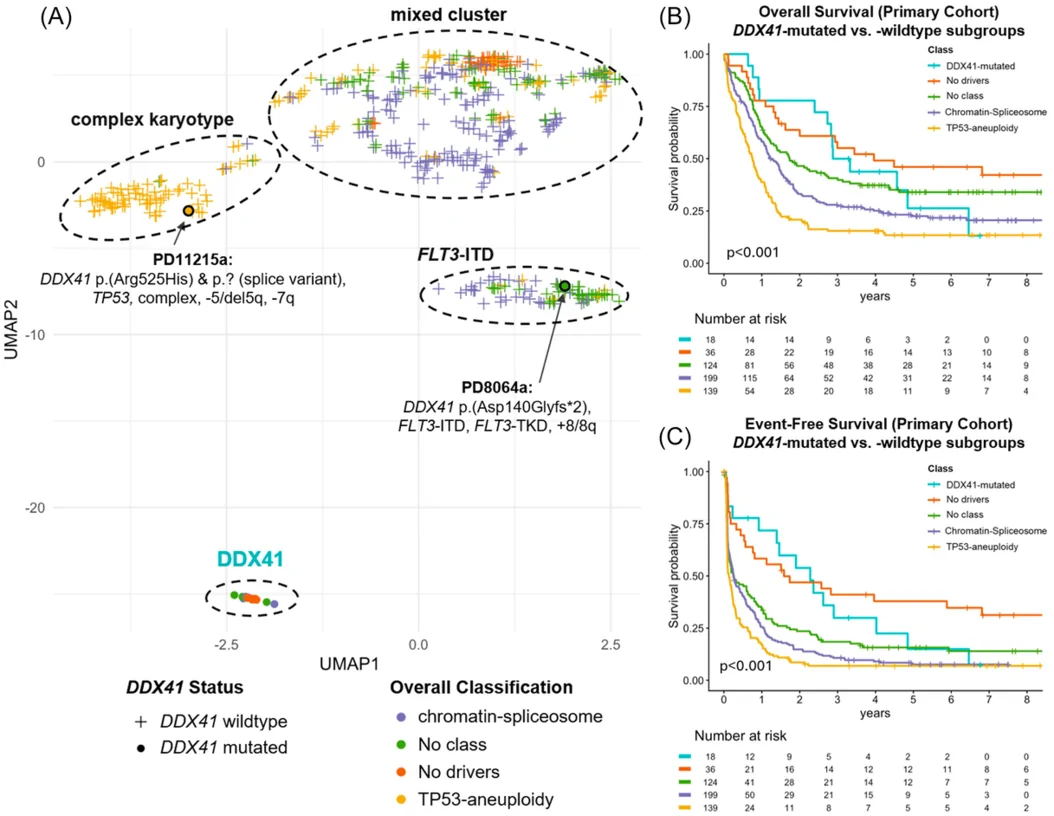
**Uniform Manifold Approximation and Projection (UMAP) clustering based on genetic features and survival outcomes of patients with *DDX41*‐mutated acute myeloid leukemia (AML) in the primary cohort of intensively treated patients (*n* = 516). (A)** Samples are color‐coded by molecular class, with *DDX41*‐mutated and ‐wildtype samples indicated by distinct symbols. Most *DDX41*‐mutated samples form a coherent cluster, whereas two *DDX41*‐mutated cases map to clusters dominated by either complex karyotype or *FLT3*‐ITD‐mutated AML (arrows). **(B)** Overall survival and event‐free survival **(C)** of patients with *DDX41*‐mutated versus *DDX41*‐wildtype AML stratified by molecular class. Survival probabilities were estimated using the Kaplan–Meier method and compared using the log‐rank test. Analyses were stratified by study, and corresponding P‐values are shown.

To derive a classification independent of predefined molecular subclasses, DBSCAN was applied to the UMAP projection. Three clusters were identified (Figure S4A). Cluster 1 (*n* = 426) included genetically heterogeneous AML, Cluster 2 (*n* = 74) was enriched for *FLT3*‐ITD, whereas Cluster 3 (*n* = 16) consisted exclusively of cases harboring pathogenic *DDX41* mutations (Tables S5 and S6). The complete segregation and internal homogeneity of this cluster further support *DDX41*‐mutated AML as a distinct molecular subgroup.

Following intensive therapy, patients with pathogenic *DDX41* variants had longer median OS than those with wildtype disease, 37.2 months (95% CI, 32.8–not reached) versus 14.2 months (95% CI, 12.9–16.7; P = 0.02; Figure S5A). Median EFS was likewise longer (27.3 vs. 2.9 months; P = 0.01; Figure S5B), whereas RFS showed a numerical but not statistically significant benefit (median, 25.8 vs. 11.5 months; P = 0.40; Figure S6A).

Relapse kinetics differed between *DDX41*‐mutated and wildtype AML, with *DDX41*‐mutated patients experiencing fewer early but more late relapses, leading to convergence of the survival curves after approximately 4–5 years. These findings should be interpreted with caution given the limited number of *DDX41*‐mutated patients who achieved remission (*n* = 15).

Compared with predefined molecular subgroups, *DDX41*‐mutated AML had longer OS than the *No_class* (20.8 months), *chromatin‐spliceosome* (15.1 months), and *TP53‐aneuploidy* (9.1 months) groups, but shorter OS than the *No_drivers* group (47.5 months; Figure 1B). Similar differences were observed for EFS and RFS (P < 0.001; Figures 1C and S6B).

In multivariable Cox regression models stratified by study and adjusted for age, sex, baseline WBC counts, AML type, and allogeneic hematopoietic stem‐cell transplantation (alloHSCT) in CR1, patients with *chromatin‐spliceosome* (hazard ratio [HR], 1.86; 95% CI, 1.03–3.37; P = 0.04) and *TP53‐aneuploidy* (HR, 3.88; 95% CI, 2.11–7.12; P < 0.001) had inferior OS compared with *DDX41*‐mutated AML. Similar results were observed for EFS (*chromatin‐spliceosome*: HR, 1.95; 95% CI, 1.12–3.39; P = 0.02; *TP53‐aneuploidy*: HR, 3.10; 95% CI, 1.76–5.46; P < 0.001), whereas RFS differed significantly only for the *TP53‐aneuploidy* group (HR, 1.95; 95% CI, 1.02–3.72; P = 0.044; Tables S3 and S4).

DBSCAN‐defined molecular clusters confirmed these findings: Cluster 3 (*DDX41*‐enriched) was independently associated with superior OS and EFS, whereas Cluster 2 showed adverse outcomes; no significant differences were observed for RFS (Figure S4B–D; Table S7).

We next sought to validate these observations in an independent cohort of 604 older patients receiving non‐intensive therapy. Pathogenic *DDX41* variants were identified in 37 patients (6.1%; Table S11). Similar to the primary cohort, patients with *DDX41*‐mutated AML were predominantly male and presented with lower WBC counts at diagnosis (Table S1).

Consistent with the primary cohort, UMAP demonstrated a clear separation of *DDX41*‐mutated AML from other molecular subgroups (Figure 2A). DBSCAN again identified a distinct *DDX41*‐enriched cluster, confirming the reproducibility of this molecular subgroup across treatment settings (Figure S7A). Only three *DDX41*‐mutated cases fell outside this cluster (Table S12). These harbored concurrent *TP53* mutation with complex karyotype, or other dominant genomic lesions and again lacked the canonical biallelic *DDX41* configuration, suggesting that these co‐occurring alterations determined the overall molecular phenotype (Table S8).

**Figure 2:**
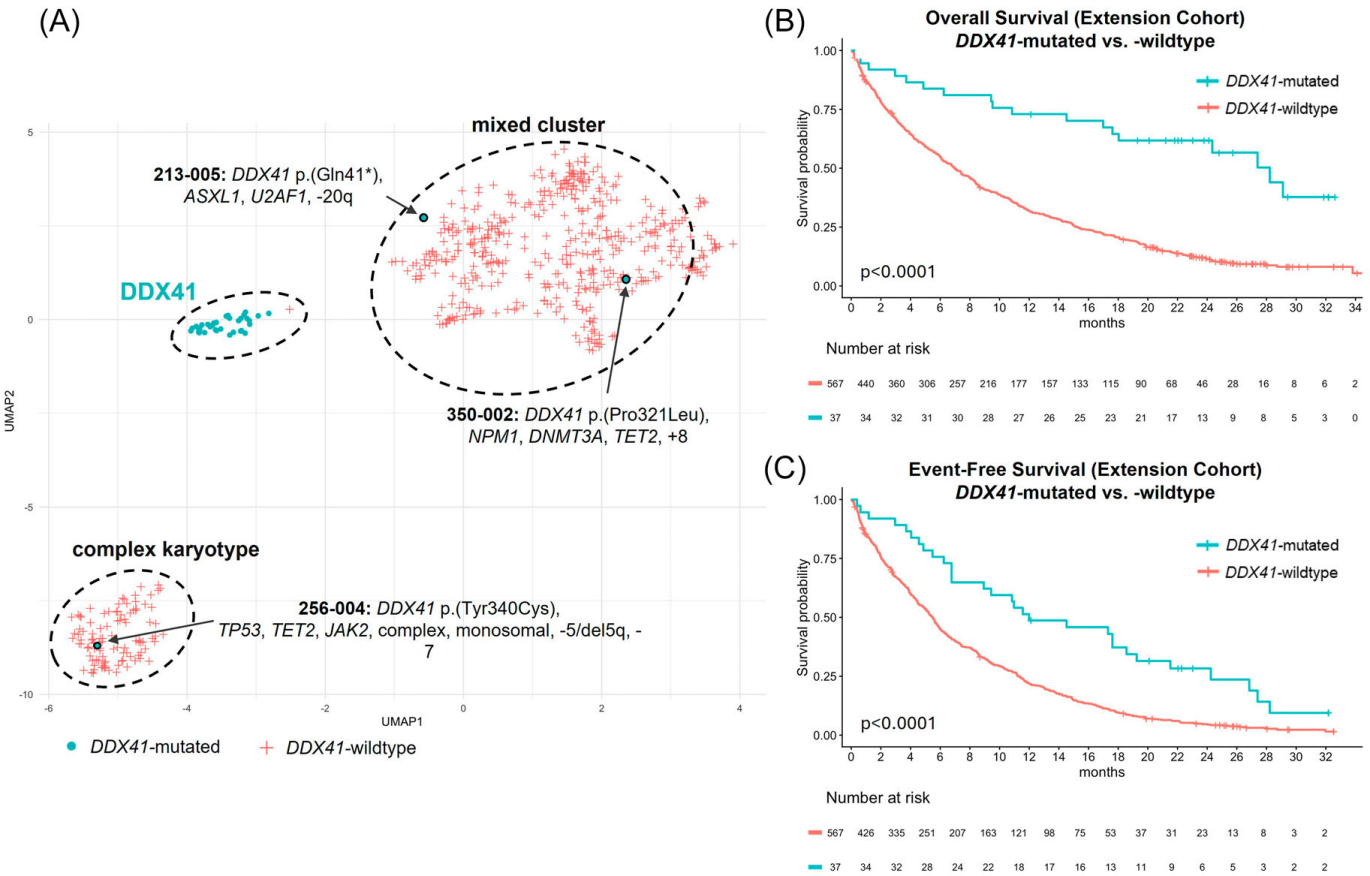
**Uniform Manifold Approximation and Projection (UMAP) clustering and survival outcomes in *DDX41*‐mutated acute myeloid leukemia (AML) in the extension cohort of non‐intensively treated patients. (A)** UMAP clustering of 604 samples based on genetic features. *DDX41*‐mutated and ‐wildtype samples are indicated as shown in the legend. *DDX41*‐mutated samples form a distinct cluster, with few exceptions. Three patients cluster outside the main *DDX41* cluster. **(B)** Overall survival and event‐free survival **(C)** of *DDX41*‐mutated compared to *DDX41*‐wildtype AML. Survival probabilities were estimated using the Kaplan–Meier method and compared using the log‐rank test; corresponding P‐values are shown.

Patients with *DDX41*‐mutated AML had significantly longer OS (median, 28.2 vs. 7.0 months; P < 0.0001) and EFS (median, 12.0 vs. 5.5 months; P < 0.0001) than *DDX41*‐wildtype patients (Figure 2B,C). In multivariable Cox regression models adjusting for age, sex, Eastern Cooperative Oncology Group (ECOG) performance status, baseline WBC, and treatment assignment, *DDX41* mutation remained independently associated with a favorable outcome (Table S3). Recapitulating the previously reported prognostic algorithm identified in the ASTRAL‐1 study, *DDX41*‐mutated AML remained the most favorable prognostic subgroup compared with *TP53*‐mutated, *FLT3*‐ITD, and “other” AML (Figure S8).^11^

Similar results were observed when patients were analyzed according to DBSCAN‐defined molecular clusters, with the *DDX41*‐enriched cluster showing the most favorable clinical outcome (Figure S7; Table S9).

In this study, we demonstrate that *DDX41*‐mutated AML constitutes a genetically coherent subgroup characterized by a unique molecular architecture and distinct clinical outcomes. Across two independent clinical trial cohorts, *DDX41*‐mutated cases consistently segregated into a discrete genomic cluster despite substantial differences in age, treatment intensity, and cohort composition. While the primary cohort was enriched for molecularly unclassified AML and excluded AML with recurrent genetic abnormalities, the reproducible clustering in an independent extension cohort supports the robustness of this observation. Nevertheless, this cohort design may have contributed to the enrichment of *DDX41*‐mutated cases within the *No_drivers* subgroup and should be considered when interpreting this finding.

The mutational spectrum closely mirrored previous reports and was characterized by frequent biallelic alterations consisting of a germline truncating variant together with a somatic hotspot mutation and few cooperating mutations.^3^, ^4^, ^6^, ^12^ In contrast, the few outlier cases clustering with *TP53*‐mutated or *FLT3*‐ITD‐positive AML lacked the canonical biallelic *DDX41* configuration, suggesting that these co‐occurring lesions determined the dominant molecular phenotype.

Consistent with previous studies, patients with *DDX41*‐mutated AML showed favorable outcomes following both intensive and non‐intensive therapy.^3^, ^5^, ^9^, ^13^, ^14^, ^15^, ^16^

However, the distinct disease trajectory characterized by favorable early disease control but an increased risk of delayed relapses, together with the notion that patients with *DDX41*‐mutated AML should preferably receive an allogeneic transplantation, argues against the classification of *DDX41*‐mutated AML as a favorable‐risk genetic subgroup in the context of intensive chemotherapy. Whether these late events represent true disease relapses or rather secondary leukemias arising on the basis of the underlying genetic predisposition currently remains unclear.^9^, ^17^, ^18^

Taken together, the reproducible genomic clustering, enrichment within the *No_drivers* subgroup, characteristic mutational profile, independent prognostic value in multivariable analyses, and distinct clinical phenotype support the recognition of *DDX41*‐mutated AML as a separate biological entity. Our findings provide additional evidence supporting its inclusion as a distinct disease entity in the forthcoming 6th edition of the WHO Classification of Haematolymphoid Tumours (WHO6).^19^

## AUTHOR CONTRIBUTIONS

**Carolin Seeling**: Investigation; visualization; writing—original draft; data curation; formal analysis; methodology. **Maral Saadati**: Visualization; data curation; investigation; formal analysis; writing—review and editing; methodology. **Daniela Späth**: Writing—review and editing; data curation. **Michael Heuser**: Writing—review and editing. **Felicitas Thol**: Writing—review and editing. **Arnold Ganser**: Writing—review and editing. **Elli Papaemmanuil**: Writing—review and editing. **Lars Bullinger**: Writing—review and editing. **Axel Benner**: Writing—review and editing; formal analysis; visualization; data curation; methodology. **Hartmut Döhner**: Conceptualization; funding acquisition; writing—original draft; investigation; resources; supervision; data curation; project administration; methodology. **Konstanze Döhner**: Data curation; supervision; resources; conceptualization; investigation; funding acquisition; writing—original draft; project administration; methodology.

## CONFLICT OF INTEREST STATEMENT

The authors declare no conflicts of interest.

## ETHICS STATEMENT

Ethics Committee of Ulm University; AMLSG BiO Registry: approval no. 148/10 (initial approval July 6, 2010; amendment no. 4, April 18, 2023), and approval no. 307/25 for the present study (December 10, 2025).

## FUNDING

This research received no funding. Open Access funding enabled and organized by Projekt DEAL.

## Supporting information

Supporting Information.

## Data Availability

The data that support the findings of this study are available in the supplementary material of this article.
