# Supplementary material for: DDX41 mutations establish a separate entity in acute myeloid leukemia with distinct survival outcomes
Source: Hemasphere. 2026 Sep 14;10(9):e70483. doi: 10.1002/hem3.70483 (PMC13574209; doi:10.1002/hem3.70483)
Supplement: Supplementary file 1 [file HEM3-10-e70483-s001.docx]

**Supplementary Appendix**

1. Supplementary Methods
   1.1. Primary Cohort of Intensively Treated Patients

The primary cohort comprised a clinically and genetically well-annotated group of 1,540 patients enrolled in three prospective multicenter clinical trials of intensive therapy conducted by the German-Austrian AML Study Group (AMLSG; AML-HD98A, AML-HD98B, and AMLSG-07-04), as previously reported by Papaemmanuil *et al.* (1). At the time, the targeted sequencing panel included 111 genes but did not cover *DDX41*, which was therefore not assessed in this cohort.

In the present study, we focused *DDX41* sequencing to subgroups outside the group of AML with recurrent genetic abnormalities: (i) AML with mutated chromatin or RNA-spliceosome genes (*chromatin-spliceosome*), (ii) AML with TP53 mutations or chromosomal aneuploidy (*TP53-aneuploidy*), (iii) AML with driver mutations but no class-defining lesions (*No_class*), and (iv) AML with no detected driver mutations (*No_drivers*).

It should be noted that these subgroup definitions were adapted from the classification proposed by Papaemmanuil *et al.* and are not part of current AML classifications (1); however, the *chromatin-spliceosome* class largely corresponds to the “AML with myelodysplasia-related gene (MRG) mutations” category of the International Consensus Classification (ICC) (2).

The *chromatin-spliceosome* subgroup was defined by mutations in genes involved in RNA splicing (*SRSF2*, *SF3B1*, *U2AF1*, and *ZRSR2*), chromatin regulation (*ASXL1*, *STAG2*, *BCOR*, *MLL*-PTD, *EZH2*, and *PHF6*), or transcription (*RUNX1*). The *TP53-aneuploidy* subgroup included cases with *TP53* mutations, complex karyotypes, cytogenetically detectable copy-number alterations (aneuploidy), or combinations thereof. The *No_class* subgroup comprised patients with identifiable driver mutations that did not meet criteria for established molecular classes (e.g., AML with *FLT3*-ITD), whereas the *No_drivers* subgroup included patients in whom no mutations were detected in any of the 111 myeloid driver genes included in the panel, potentially reflecting genomic alterations not captured by the assay (1).

Among the 702 patients assigned to these subgroups, DNA specimens suitable for *DDX41* sequencing were available for 516 individuals. The median age of these patients was 53.5 years (range 18-84 years), and all received intensive chemotherapy (**Suppl. Table 1**). Median follow-up was 6.4 years (95% CI 5.7-6.8).

1.2. Extension Cohort of Non-Intensively Treated Patients

Genomic and clinical data were obtained from a prospective, randomized trial (NCT02348489; ASTRAL-1), in which patients with previously untreated AML unfit for intensive chemotherapy were randomized to guadecitabine or treatment choice (azacitidine, decitabine, or low-dose cytarabine). Of the 815 patients included in the clinical trial, 604 provided consent for molecular analyses and were included in this study (3, 4). Importantly, patients in this cohort were not pre-selected based on molecular subgroup, providing an unbiased population for *DDX41* analysis.

Genomic profiling was previously performed using a 263-gene sequencing panel, which included *DDX41*. Certain *DDX41* germline variants had not been detected by the variant-calling pipeline used in the original publication (3). A retrospective review of all *DDX41* variants, including manual curation of sequencing data, enabled the identification of five additional variants, whereas one *DDX41* variant was reclassified from pathogenic to a variant of uncertain significance (VUS) according to updated pathogenicity criteria, resulting in a total of 37 patients with pathogenic *DDX41* variants.

For univariate and multivariate analyses, patients were stratified into four molecular subgroups: *DDX41*-mutated, *TP53*-mutated, *FLT3* internal tandem duplications (*FLT3*-ITD), and “other” (defined as the absence of the aforementioned alterations), based on prognostic stratification previously reported for this trial (3).

The median age of the patients was 77.0 years (range 59-94 years). The median follow-up was 2.2 years (95% CI 2.1-2.3). Baseline patient and disease characteristics are summarized in **Suppl. Table 1**.

1.3. Gene Mutation Analysis of the Primary Cohort

Targeted sequencing of *DDX41* was performed across the entire coding region using a custom single-gene panel (Twist Bioscience). Libraries were prepared from 100 ng DNA and sequenced with 2 x 150 bp paired-end reads on a NextSeq 500 (Illumina). Median read depth was 4,087x (range 64-9,875x). Sequencing data were analyzed with varvis® v2.1.0 and variants with a VAF ≥1% were considered. To ensure methodological consistency with the previously published ASTRAL-1 extension cohort included in this study (3), *DDX41* variants with a VAF ≥38%, suggestive of a possible germline origin, and a population frequency below 1% (according to the Genome Aggregation Database), were selected and classified according to the American College of Medical Genetics and Genomics (ACMG) and the Association for Molecular Pathology (5). A VAF threshold of ≥38%, retained from the ASTRAL-1 extension cohort rather than applying the conventional 40% threshold, was used because recurrent *DDX41* variants with established germline association may present with VAFs slightly below 40%. In accordance with ACMG recommendations, VAF was considered supportive rather than definitive evidence of germline origin, as no validated VAF threshold reliably distinguishes germline from somatic variants (6).

Additional genomic data were obtained from existing molecular datasets of the corresponding cohorts (1, 3).

Diagnostic sequencing was performed on either bone marrow (BM) or peripheral blood (PB) samples according to sample availability at diagnosis. Sequencing methodology and read depth were comparable between BM- and PB-derived samples. All cases classified as *DDX41* germline-only mutated (n = 5; **Suppl. Figure 1**) were analyzed using PB samples. Therefore, low-frequency somatic *DDX41* second-hit mutations may have remained below the assay’s limit of detection.

1.4. Statistical Analyses

High-dimensional mutation profiles were embedded into two-dimensional space using Uniform Manifold Approximation and Projection (UMAP). Clusters of similar samples were then identified with Density-Based Spatial Clustering of Applications with Noise (DBSCAN), with unassigned samples labeled as noise (7). Overall survival (OS), event-free survival (EFS), and relapse-free survival (RFS) were estimated using the Kaplan-Meier method, p-values were calculated using stratified (by study) log-rank tests. Multivariate Cox regression models stratified by study were adjusted for age, sex, ECOG performance status, baseline white blood cell count (WBC), treatment, allogeneic hematopoietic stem-cell transplantation in first complete remission (alloHSCT in CR1), and molecular/prognostic class or cluster membership, as appropriate.

Missing data were imputed with multiple imputation by chained equations (MICE) to generate m = 1 imputed data set. Analyses were conducted in R (version 4.5.1). Mutation landscapes were visualized using ProteinPaint (St. Jude Children’s Research Hospital) (8).

In the primary cohort, a total of 69 genetic variables, including mutation variables and cytogenetic aberrations, were included. Of note, in the ASTRAL-1 extension cohort, the sequencing panel was substantially larger (263 genes); to ensure comparability and to minimize instability related to very low-frequency events during imputation, only genetic variants (gene mutations and cytogenetic aberrations) detected with a prevalence greater than 3% across patients were retained, resulting in 52 variables.

1.5. References

1.	Papaemmanuil E, Gerstung M, Bullinger L, Gaidzik VI, Paschka P, Roberts ND, et al. Genomic Classification and Prognosis in Acute Myeloid Leukemia. N Engl J Med. 2016;374(23):2209-21.

2.	Arber DA, Orazi A, Hasserjian RP, Borowitz MJ, Calvo KR, Kvasnicka HM, et al. International Consensus Classification of Myeloid Neoplasms and Acute Leukemias: integrating morphologic, clinical, and genomic data. Blood. 2022;140(11):1200-28.

3.	Jahn E, Saadati M, Fenaux P, Gobbi M, Roboz GJ, Bullinger L, et al. Clinical impact of the genomic landscape and leukemogenic trajectories in non-intensively treated elderly acute myeloid leukemia patients. Leukemia. 2023;37(11):2187-96.

4.	Fenaux P, Gobbi M, Kropf PL, Issa JJ, Roboz GJ, Mayer J, et al. Guadecitabine vs treatment choice in newly diagnosed acute myeloid leukemia: a global phase 3 randomized study. Blood Adv. 2023;7(17):5027-37.

5.	Richards S, Aziz N, Bale S, Bick D, Das S, Gastier-Foster J, et al. Standards and guidelines for the interpretation of sequence variants: a joint consensus recommendation of the American College of Medical Genetics and Genomics and the Association for Molecular Pathology. Genet Med. 2015;17(5):405-24.

6.	Li MM, Chao E, Esplin ED, Miller DT, Nathanson KL, Plon SE, et al. Points to consider for reporting of germline variation in patients undergoing tumor testing: a statement of the American College of Medical Genetics and Genomics (ACMG). Genet Med. 2020;22(7):1142-8.

7.	Ester M, Kriegel HP, Sander J, Xu X. A density-based algorithm for discovering clusters in large spatial databases with noise. In: Proceedings of the Second International Conference on Knowledge Discovery and Data Mining (KDD-96). Portland, OR: AAAI Press; 1996. p. 226-31.

8.	Zhou X, Edmonson MN, Wilkinson MR, Patel A, Wu G, Liu Y, et al. Exploring genomic alteration in pediatric cancer using ProteinPaint. Nat Genet. 2016;48(1):4-6.

2. Supplementary Figures & Tables

**Suppl. Figure 1: Genetic architecture of DDX41-mutated AML in the primary cohort.** (**A**) Mutational landscape of DDX41. Variants are mapped relative to the protein: germline variants above the axis, somatic variants below. Variants are color-coded: truncating (red), non-truncating (blue), and uncertain germline (green). Symbol size indicates the number of samples harboring the variant. (**B**) Oncoplot of DDX41-mutated samples showing the most frequent co-mutations and cytogenetic alterations. Colors indicate molecular class, and sample origin is indicated as bone marrow (BM) or peripheral blood (PB).

**Suppl. Figure 2: Heatmap of mutation and cytogenetic alteration frequencies in the primary cohort.** Heatmap showing the frequency of mutations and cytogenetic aberrations in the primary cohort (n=516). The dataset includes 69 genes and cytogenetic abnormalities. Missing values are represented as blank (white) spaces.

**Suppl. Figure 3:** **UMAP visualization of mutation status for all analyzed genes**. Uniform Manifold Approximation and Projection (UMAP) plots for all genes analyzed, with samples colored according to mutation status (turquoise=mutated, red=wildtype).

**Suppl. Figure 4:** **DBSCAN clustering after UMAP projection and survival outcomes in the primary cohort.** (**A**) DBSCAN clustering after UMAP projection in the primary cohort. DDX41-mutated and -wildtype samples are indicated by symbols, and clusters are color-coded according to the assigned DBSCAN cluster. Overall survival (OS; **B**) and event-free survival (EFS; **C**) and relapse-free survival (RFS; **D**) are shown by cluster.

**Suppl. Figure 5: Survival outcomes in DDX41-mutated AML in the primary cohort of intensively treated patients**. Overall survival (**A**) and event-free survival (**B**) of patients with DDX41-mutated versus DDX41-wildtype AML. Survival probabilities were estimated using the Kaplan-Meier method and compared using the log-rank test. Analyses were stratified by study, and corresponding p-values are shown.

**Suppl. Figure 6: Relapse-free survival (RFS) in DDX41-mutated AML in the primary cohort.** (**A**) RFS of patients with DDX41-mutated versus DDX41-wildtype AML (**B**) RFS with DDX41-mutated patients shown as one group, while DDX41-wildtype patients are further stratified by molecular class. Survival probabilities were estimated using the Kaplan-Meier method and compared using the log-rank test. Analyses were stratified by study, and corresponding p-values are shown.

**Suppl. Figure 7:** **DBSCAN clustering after UMAP projection with survival outcomes by cluster in the extension cohort.** (**A**) DBSCAN clustering after UMAP projection in the extension cohort. DDX41-mutated and -wildtype samples are indicated by symbols, and clusters are color-coded according to the assigned DBSCAN cluster. Overall survival (OS; **B**) and event-free survival (EFS; **C**) are shown by clusters.

**Suppl. Figure 8:** **Survival of DDX41-mutated AML compared with prognostic subgroups of DDX41-wildtype AML in the extension cohort.** (**A**) Overall survival and (**B**) event-free survival of DDX41-mutated compared with DDX41-wildtype AML, with DDX41-wildtype patients further stratified into prognostic subgroups. Survival probabilities were estimated using the Kaplan-Meier method and compared using the log-rank test; corresponding p-values are shown.

**Suppl. Table 1:** **Baseline characteristics of younger intensively treated patients (primary cohort) and older non-intensively treated patients (extension cohort).**
Abbr: alloHSCT – allogeneic hematopoietic stem-cell transplantation, ATRA – All-Trans Retinoic Acid, ECOG – Eastern Cooperative Oncology Group performance status, ICE -Idarubicin/Cytarabine/Etoposide, LDAC – Low-Dose Cytarabine, Mut – Mutant, SD – Standard Deviation, WBC – White Blood Cell Count, WT – Wildtype

|  | Primary Cohort (AMLSG Trials) | | | Extension Cohort (ASTRAL-1 Trial) | | |
| --- | --- | --- | --- | --- | --- | --- |
|  | **Total (n=516)** | ***DDX41*-WT (n=498)** | ***DDX41*-Mut (n=18)** | **Total (n=604)** | ***DDX41*-WT (n=567)** | ***DDX41*-Mut (n=37)** |
| Median Age [Min, Max] | 53.5 [18.0, 84.0] | 53.0 [18.0, 84.0] | 58.5 [29.0, 79.0] | 77.0 [59.0, 94.0] | 77.0 [59.0, 94.0] | 75.0 [61.0, 86.0] |
| Sex  Female  Male | 208 (40.3%)  308 (59.7%) | 204 (41.0%)  294 (59.0%) | 4 (22.2%)  14 (77.8%) | 255 (42.2%)  349 (57.8%) | 244 (43.0%)  323 (57.0%) | 11 (29.7%)  26 (70.3%) |
| WBC x10^9^/L  Mean (SD)  Median [Min, Max]  Missing | 32.7 (52.9)  8.25 [0.400, 427]  6 (1.2%) | 33.6 (53.5)  9.20 [0.400, 427]  6 (1.2%) | 8.94 (21.0)  2.10 [0.700, 73.2]  0 (0%) | 11.5 (21.1)  3.59 [0.300, 216]  0 (0%) | 12.1 (21.6)  3.89 [0.300, 216]  0 (0%) | 2.17 (2.55)  1.71 [0.500, 16.5]  0 (0%) |
| ECOG  0  1  2  3  4  Missing | 106 (20.5%)  280 (54.3%)  53 (10.3%)  6 (1.2%)  2 (0.4%)  69 (13.4%) | 98 (19.7%)  271 (54.4%)  53 (10.6%)  6 (1.2%)  2 (0.4%)  68 (13.7%) | 8 (44.4%)  9 (50.0%)  0 (0%)  0 (0%)  0 (0%)  1 (5.6%) | 77 (12.7%)  216 (35.8%)  250 (41.4%)  61 (10.1%)  0 (0%)  0 (0%) | 71 (12.5%)  194 (34.2%)  243 (42.9%)  59 (10.4%)  0 (0%)  0 (0%) | 6 (16.2%)  22 (59.5%)  7 (18.9%)  2 (5.4%)  0 (0%)  0 (0%) |
| AlloHSCT in CR1  Yes  No  Missing | 123 (23.8%)  392 (76.0%)  1 (0.2%) | 118 (23.7%)  380 (76.3%)  0 (0%) | 5 (27.8%)  12 (66.7%)  1 (5.6%) | -  -  - | -  -  - | -  -  - |
| AlloHSCT Overall  Yes  No  Missing/Donor type unknown | 236 (45.7%)  277 (53.7%)  3 (0.6%) | 225 (45.2%)  270 (54.2%)  3 (0.6%) | 11 (61.1%)  7 (38.9%)  0 (0%) | -  -  - | -  -  - | -  -  - |
| Intensive Therapy  ICE  ATRA-ICE | 384 (74.4%)  132 (25.6%) | 373 (74.9%)  125 (25.1%) | 11 (61.1%)  7 (38.9%) | -  - | -  - | -  - |
| Non-intensive Therapy  Guadecitabine  Azacitidine  Decitabine  LDAC  No Treatment | -  -  -  -  - | -  -  -  -  - | -  -  -  -  - | 290 (48%)  154 (25%)  124 (21%)  21 (3.5%)  15 (2.5%) | 270 (47.6%)  145 (25.6%)  117 (20.6%)  21 (3.7%)  14 (2.5%) | 20 (54.1%)  9 (24.3%)  7 (18.9%)  0 (0%)  1 (2.7%) |

**Suppl. Table 2: Pairwise comparison of pathogenic DDX41 variant frequencies between molecular classes.** Pairwise Fisher’s exact tests were used to compare DDX41 frequencies across classes (chromatin-spliceosome, No_class, No_drivers, TP53-aneuploidy). P-values were Holm-adjusted. The table shows sample number (n), raw p-values (p), adjusted p-values (p.adj), and significance levels.

| group1 | group2 | n | p | p.adj | p.adj.signif |
| --- | --- | --- | --- | --- | --- |
| *chromatin-spliceosome* | *No_class* | 331 | 0.492 | 0.984 | ns |
| *chromatin-spliceosome* | *No_drivers* | 248 | <0.0001 | <0.001 | *** |
| *chromatin-spliceosome* | *TP53-aneuploidy* | 343 | 0.652 | 0.984 | ns |
| *No_class* | *No_drivers* | 173 | <0.001 | 0.003 | ** |
| *No_class* | *TP53-aneuploidy* | 268 | 0.196 | 0.588 | ns |
| *No_drivers* | *TP53-aneuploidy* | 185 | <0.0001 | <0.0001 | **** |

**Suppl. Table 3: Multivariate analysis of overall survival and event-free survival in the primary and extension cohorts.** Abbr: 95% CI – 95% Confidence Interval, alloHSCT – allogeneic hematopoietic stem-cell transplantation, CR – complete remission, HR – Hazard Ratio, ITD – Internal Tandem Duplication, sAML – secondary AML, Sign. – Significance, tAML – therapy-related AML, WBC – white blood cell count, yr – year(s)

| Cohort of younger, intensively treated patients | | | | | | | | |
| --- | --- | --- | --- | --- | --- | --- | --- | --- |
|  | **Overall Survival** | | | | **Event-Free Survival** | | | |
| Variable | **HR** | **95*%* CI** | **p-value** | **Sign.** | **HR** | **95% CI** | **p-value** | **Sign.** |
| Age_10yr | 1.28 | [1.15;1.43] | <0.001 | *** | 1.14 | [1.04;1.25] | 0.005 | ** |
| Sex  female  male | Ref  1.25 | [1.01;1.54] | 0.041 | * | Ref  1.34 | [1.10;1.62] | 0.004 | ** |
| Type of AML  de novo  sAML/tAML | Ref  1.27 | [0.92;1.76] | 0.152 |  | Ref  1.03 | [0.76;1.39] | 0.865 |  |
| WBC x10^9^/L | 1.56 | [1.32;1.83] | <0.001 | *** | 1.29 | [1.11;1.49] | <0.001 | *** |
| AlloHSCT in CR1 | 0.77 | [0.57;1.03] | 0.077 |  | 0.58 | [0.39;0.85] | 0.006 | ** |
| Molecular Class  *DDX41*-mutated  No_drivers  No_class  chromatin-spliceosome  *TP53*-aneuploidy | Ref  0.98  1.50  1.86  3.88 | [0.48;2.00]  [0.80;2.82]  [1.03;3.37]  [2.11;7.12] | 0.956  0.208  0.041  <0.001 | *  *** | Ref  0.88  1.79  1.95  3.10 | [0.45;1.70]  [0.99;3.21]  [1.12;3.39]  [1.76;5.46] | 0.699  0.053  0.018  <0.001 | *  *** |
| Cohort of older, non-intensively treated patients | | | | | | | | |
|  | **Overall Survival** | | | | **Event-Free Survival** | | | |
| Variable | **HR** | **95*%* CI** | **p-value** | **Sign.** | **HR** | **95% CI** | **p-value** | **Sign.** |
| Age_10yr | 1.25 | [1.06;1.47] | 0.007 | ** | 1.17 | [1.00;1.37] | 0.043 | * |
| Sex  female  male | Ref  1.27 | [1.06;1.52] | 0.009 | ** | Ref  1.30 | [1.10;1.54] | 0.003 | ** |
| Treatment  Guadecitabine  Treatment Choice | Ref  1.10 | [0.92;1.30] | 0.295 |  | Ref  0.98 | [0.83;1.15] | 0.786 |  |
| WBC x10^9^/L | 1.57 | [1.32;1.87] | <0.001 | *** | 1.42 | [1.19;1.69] | <0.001 | *** |
| ECOG ≥2 | 1.49 | [1.23;1.80] | <0.001 | *** | 1.30 | [1.09;1.56] | 0.004 | ** |
| Prognostic Group  *DDX41*-mutated  *TP53*-mutated  *FLT3*-ITD  Other | Ref  3.79  3.98  2.42 | [2.28;6.31]  [2.25;7.03]  [1.49;3.92] | <0.001 <0.001  <0.001 | ***  ***  *** | Ref  2.31  2.67  1.65 | [1.53;3.49]  [1.64;4.35]  [1.12;2.43] | <0.001 <0.001  0.011 | ***  ***  * |

**Suppl. Table 4: Multivariate analysis of relapse-free survival in the primary cohort.** Abbr: 95% CI – 95% Confidence Interval, alloHSCT – allogeneic hematopoietic stem-cell transplantation, CR – complete remission, HR – Hazard Ratio, sAML – secondary AML, Sign. – Significance, tAML – therapy-related AML, WBC – white blood cell count, yr – year(s)

|  | Relapse-Free Survival | | | |
| --- | --- | --- | --- | --- |
| Variable | **HR** | **95*%* CI** | **p-value** | **Sign.** |
| Age_10yr | 1.13 | [1.00;1.26] | 0.046 | * |
| Sex  female  male | Ref  1.16 | [0.91;1.48] | 0.241 |  |
| Type of AML  de novo  sAML/tAML | Ref  1.26 | [0.84;1.90] | 0.266 |  |
| WBC log10 | 1.49 | [1.23;1.80] | <0.001 | *** |
| AlloHSCT in CR1 | 0.66 | [0.49;0.89] | 0.006 | ** |
| Molecular Class  *DDX41*-mutated  No_drivers  No_class  chromatin-spliceosome  *TP53*-aneuploidy | Ref  0.65  0.97  1.35  1.95 | [0.31;1.36]  [0.50;1.89]  [0.73;2.51]  [1.02;3.72] | 0.254  0.931  0.342  0.044 | * |

**Suppl. Table 5: DBSCAN-derived clusters in the two cohorts and their most frequent genetic alterations (frequency).**

| Primary Cohort | |
| --- | --- |
| Cluster | **Alterations (frequency)** |
| 1 | RUNX1 (17%), minus5_5q (15%), NRAS (15%), DNMT3A (14%), TP53 (14%), SRSF2 (12%), IDH2 (12%), minus7 (11%), TET2 (10%), MLL-PTD (9%) |
| 2 | FLT3_ITD (99%), DNMT3A (19%), MLL (19%), RUNX1 (15%), TET2 (12%), CEBPA (11%), IDH1 (8%), WT1 (8%), FLT3_TKD (7%), FLT3_other (7%) |
| 3 | DDX41 (100%), ASXL1 (19%), PHF6 (19%), SRSF2 (12%), minus7q (6%), DNMT3A (6%), EZH2 (6%), NRAS (6%), RUNX1 (6%) |
| Extension Cohort | |
| Cluster | **Alterations (frequency)** |
| 1 | ASXL1 (32%), TET2 (31%), SRSF2 (28%), RUNX1 (26%), DNMT3A (24%), NPM1 (20%), STAG2 (16%), NRAS (15%), +8 (14%), complex.ELN2022 (13%) |
| 2 | complex.ELN2022 (100%), TP53 (94%), -5/del5q (92%), monosomal (80%), -17/17p-/abn(17) (57%), -7 (43%), +8 (34%), 12p- (28%), 3p- (27%), 7q- (27%) |
| 3 | DDX41 (97%), ASXL1 (26%), DNMT3A (17%), EZH2 (14%), TET2 (14%), complex.ELN2022 (6%), CSMD1 (6%), PHF6 (6%), SRSF2 (6%), 7q- (3%) |

**Suppl. Table 6: DDX41-mutated patients in DBSCAN-derived clusters and their co-mutations in the primary cohort.**

| Cluster | Patient-ID | Co-Mutations |
| --- | --- | --- |
| 1 | PD11215a | minus5_5q, minus7q, complex, TP53, DDX41 |
| 2 | PD8064a | plus8_8q, FLT3_TKD, FLT3_ITD, DDX41 |
| 3 | PD10888a | DDX41 |
| 3 | PD11060a | ASXL1, EZH2, PHF6, DDX41 |
| 3 | PD11130a | DDX41 |
| 3 | PD11149a | DDX41 |
| 3 | PD11191a | PHF6, DDX41 |
| 3 | PD11209a | ASXL1, NRAS, SRSF2, DDX41 |
| 3 | PD11270a | DDX41 |
| 3 | PD7758a | DDX41 |
| 3 | PD7785a | DDX41 |
| 3 | PD7960a | DDX41 |
| 3 | PD8136a | plus8_8q, PHF6, DDX41 |
| 3 | PD8155a | DNMT3A, DDX41 |
| 3 | PD8261a | SRSF2, DDX41 |
| 3 | PD8345a | minus7q, ASXL1, RUNX1, DDX41 |
| 3 | PD8402a | DDX41 |
| 3 | PD8428a | DDX41 |

**Suppl. Table 7: Multivariate analysis of overall survival, event-free survival, and relapse-free survival using DBSCAN clustering in the primary cohort.** Abbr: 95*%* CI – 95% Confidence Interval, alloHSCT – allogeneic hematopoietic stem-cell transplantation, CR – complete remission, HR – Hazard Ratio, sAML – secondary AML, Sign. – Significance, tAML – therapy-related AML, WBC – white blood cell count, yr – year(s)

|  | Overall Survival | | | | Event-Free Survival | | | | Relapse-Free Survival | | | |
| --- | --- | --- | --- | --- | --- | --- | --- | --- | --- | --- | --- | --- |
| Variable | **HR** | **95*%* CI** | **p-value** | **Sign.** | **HR** | **95% CI** | **p-value** | **Sign.** | **HR** | **95*%* CI** | **p-value** | **Sign.** |
| Age_10yr | 1.30 | [1.17;1.45] | <0.001 | *** | 1.17 | [1.06;1.28] | 0.001 | ** | 1.15 | [1.02;1.29] | 0.018 | * |
| Sex  female  male | Ref  1.32 | [1.07;1.63] | 0.0099 | ** | Ref  1.38 | [1.13;1.67] | 0.001 | ** | Ref  1.19 | [0.93;1.52] | 0.166 | ** |
| Type of AML  de novo  sAML/tAML | Ref  1.42 | [1.03;1.97] | 0.035 |  | Ref  1.21 | [0.89;1.63] | 0.227 |  | Ref  1.44 | [0.95;2.17] | 0.084 |  |
| WBC log10 | 1.45 | [1.24;1.70] | <0.001 | *** | 1.29 | [1.12;1.49] | <0.001 | *** | 1.48 | [1.23;1.80] | <0.001 | *** |
| AlloHSCT in CR1 | 0.87 | [0.65;1.15] | 0.322 |  | 0.58 | [0.40;0.85] | 0.004 | ** | 0.72 | [0.54;0.96] | 0.027 | * |
| Cluster  1  2  3 | Ref  1.16  0.44 | [0.86;1.57]  [0.24;0.83] | 0.324  0.011 | * | Ref  1.12  0.51 | [0.84;1.48]  [0.29;0.89] | 0.448  0.018 | * | Ref  1.05  0.74 | [0.74;1.49]  [0.40;1.39] | 0.792  0.352 |  |

**Suppl. Table 8: DDX41-mutated patients in DBSCAN-derived clusters and their co-mutations in the extension cohort.**

| Cluster | Patient-ID | Co-Mutations |
| --- | --- | --- |
| 1 | 213-005 | 20q-, ASXL1, U2AF1, DDX41 |
| 1 | 350-002 | +8, DNMT3A, NPM1, TET2, DDX41 |
| 2 | 256-004 | complex.ELN2022, monosomal, -5/del5q, -7, JAK2, TET2, TP53, DDX41 |
| 3 | 101-012 | DNMT3A, EZH2, DDX41 |
| 3 | 102-004 | ASXL1, DDX41 |
| 3 | 102-007 | 20q-, DDX41 |
| 3 | 104-002 | DDX41 |
| 3 | 116-004 | ASXL1, DDX41 |
| 3 | 154-002 | complex.ELN2022, +8, -17/17p-/abn(17), DNMT3A, DDX41 |
| 3 | 215-004 | DDX41 |
| 3 | 215-014 | DDX41 |
| 3 | 218-020 | DDX41 |
| 3 | 219-002 | CSMD1, SF3B1, SRSF2, DDX41 |
| 3 | 219-003 | ASXL1, TET2, DDX41 |
| 3 | 219-008 | DNMT3A, EZH2, DDX41 |
| 3 | 220-001 | SRSF2, DDX41 |
| 3 | 230-001 | DDX41 |
| 3 | 230-006 | TP53, DDX41 |
| 3 | 230-009 | DDX41 |
| 3 | 234-002 | DDX41 |
| 3 | 234-003 | ASXL1, DNMT3A, NRAS, DDX41 |
| 3 | 238-001 | DDX41 |
| 3 | 240-006 | DDX41 |
| 3 | 256-005 | complex.ELN2022, 13q-, CSMD1, U2AF1, DDX41 |
| 3 | 256-015 | 7q-, TET2, DDX41. |
| 3 | 271-004 | ASXL1, EZH2, DDX41 |
| 3 | 280-006 | ASXL1, EZH2, DDX41 |
| 3 | 286-002 | PHF6, DDX41 |
| 3 | 286-004 | DNMT3A, DDX41 |
| 3 | 301-001 | ASXL1, DDX41 |
| 3 | 302-005 | TET2, DDX41 |
| 3 | 303-001 | DDX41 |
| 3 | 312-020 | ASXL1, PHF6, DDX41 |
| 3 | 331-005 | TET2, DDX41 |
| 3 | 346-001 | CUX1, TET2, DDX41 |
| 3 | 346-011 | DNMT3A, DDX41 |
| 3 | 402-008 | DDX41 |

**Suppl. Table 9:** **Multivariate analysis of overall survival and event-free survival using DBSCAN clustering in the extension cohort.** Abbr: 95% CI – 95% Confidence Interval, ECOG – Eastern Cooperative Oncology Group performance status, GUA – Guadecitabine, HR – Hazard Ratio, Sign. – Significance, WBC – white blood cell count, yr – year(s)

|  | Overall Survival | | | | Event-Free Survival | | | |
| --- | --- | --- | --- | --- | --- | --- | --- | --- |
| Variable | **HR** | **95*%* CI** | **p-value** | **Sign.** | **HR** | **95% CI** | **p-value** | **Sign.** |
| Treatment  GUA  Treatment Choice | Ref  1.09 | [0.92;1.30] | 0.31 |  | Ref  0.97 | [0.82;1.14] | 0.691 |  |
| Age_10yr | 1.26 | [1.07;1.49] | 0.005 | ** | 1.17 | [1.01;1.37] | 0.04 | * |
| Sex  female  male | Ref  1.26 | [1.05;1.50] | 0.011 | * | Ref  1.27 | [1.07;1.51] | 0.006 | ** |
| ECOG ≥2 | 1.46 | [1.21;1.76] | <0.001 | *** | 1.29 | [1.07;1.54] | 0.006 | ** |
| WBC log10 | 1.63 | [1.38;1.94] | <0.001 | *** | 1.48 | [1.25;1.75] | <0.001 | *** |
| Cluster  1  2  3 | Ref  1.52  0.35 | [1.22;1.90]  [0.21;0.59] | <0.001  <0.001 | ***  *** | Ref  1.36  0.57 | [1.09;1.70]  [0.38;0.84] | 0.006  0.005 | **  ** |

**Suppl. Table 10:** **Molecular and cytogenetic profiles of individuals with DDX41 variants in the primary cohort.** Abbr: ITD – Internal Tandem Duplication, TKD – Tyrosine Kinase Domain, VAF – Variant Allele Frequency

| **Pathogenic *DDX41* Variants** | | | | | | | | | |
| --- | --- | --- | --- | --- | --- | --- | --- | --- | --- |
| **Patient** | **Class** | **germline *DDX41*** | | | **somatic *DDX41*** | | | **Concomitant variants** | **Cytogenetics** |
|  |  | **Codon** | **Protein** | **VAF** | **Codon** | **Protein** | **VAF** |  |  |
| PD7758a | No drivers | c.121C>T | p.(Gln41*) | 48% | c.1574G>A | p.(Arg525His) | 7% |  | 46,XY |
| PD7785a | No drivers | c.1169T>C | p.(Leu390Pro) | 48% | c.1574G>A | p.(Arg525His) | 1% |  | 46,XY |
| PD11270a | No drivers | c.571G>A | p.(Ala191Thr) | 47% | c.1574G>A | p.(Arg525His) | 6% |  | 46,XY |
| PD8402a | No drivers | c.1085_1087del | p.(Phe362del) | 48% | c.1574G>A | p.(Arg525His) | 3% |  | no metaphases |
| PD8428a | No drivers | c.3G>A | p.(Met1Ile) | 46% | c.1574G>A | p.(Arg525His) | 2% |  | 46,XY |
| PD10888a | No drivers | c.3G>A | p.(Met1Ile) | 51% |  |  |  |  | 46,XX |
| PD7960a | No drivers | c.1154_1158del | p.(Phe385*) | 44% |  |  |  |  | 46,XX |
| PD11149a | No drivers | c.415_418dup | p.(Asp140Glyfs*2) | 43% |  |  |  |  | 46,XY |
| PD11130a | No drivers |  |  |  | c.1574G>A | p.(Arg525His) | 1% |  | 46,XY,inv(9)(p11q12)c[2]/46,XY[3] |
| PD11191a | No class | c.931C>T | p.(Arg311*) | 47% | c.1574G>A | p.(Arg525His) | 9% | PHF6 (c.941T>C, p.(Ile314Thr), VAF 18%) | 46,XY |
| PD8064a | No class | c.415_418dup | p.(Asp140Glyfs*2) | 44% |  |  |  | FLT3-ITD | 47,XX,+8 |
|  |  |  |  |  |  |  |  | FLT3-TKD (p.(Asp835)) |  |
| PD8136a | No class | c.435-2_435-1delinsCA | p.? (Splicing Variant) | 47% | c.1574G>A | p.(Arg525His) | 15% | PHF6 (c.824G>T, p.(Gly275Val), VAF 31%) | 47,XY,+8[4]/46,XY[13] |
| PD8155a | No class | c.3G>A | p.(Met1Ile) | 50% | c.1574G>A | p.(Arg525His) | 2% | DNMT3A (c.2207G>A, p.(Arg736His), VAF 18%) | no metaphases |
| PD8261a | chromatin_ spliceosome | c.3G>A | p.(Met1Ile) | 48% | c.1574G>A | p.(Arg525His) | 14% | SRSF2 (c.284C>A, p.(Pro95His), VAF 6%) | 46,XY,inv(9)(p13q22) |
| PD8345a | chromatin_ spliceosome | c.992_994del | p.(Lys331del) | 43% | c.1574G>A | p.(Arg525His) | 44% | ASXL1 (c.2381_2384delCTTC, p.(Ser795fs*22), VAF 49%) | 46,XY,del(7)(q22)[10]/46,XY[2] |
|  |  |  |  |  |  |  |  | RUNX1 (c.930_931insGCTCG, p.(Thr311fs*19), VAF 33%) |  |
| PD11060a | chromatin_ spliceosome | c.435-2_435-1delinsCA | p.? (Splicing Variant) | 46% | c.1589G>A | p.(Gly530Asp) | 26% | ASXL1 (c.2485C>T, p.(Gln829*), VAF 16%) | 46,XY[20] |
|  |  |  |  |  |  |  |  | EZH2 (c.391A>T, p.(Ile131Phe), VAF 9%) |  |
|  |  |  |  |  |  |  |  | PHF6 (c.5C>G, p.(Ser2*), VAF 7%) |  |
| PD11209a | chromatin_ spliceosome | c.934C>T | p.(His312Tyr) | 50% |  |  |  | ASXL1 (c.1762C>T, p.(Gln588*), VAF 47%) | 46,XY |
|  |  |  |  |  |  |  |  | NRAS (c.34G>T, p.(Gly12Cys), VAF 40%) |  |
|  |  |  |  |  |  |  |  | SRSF2 (c.284C>G, p.(Pro95Arg), VAF 48%) |  |
| PD11215a | p53_ aneuploidy |  |  |  | c.1574G>A | p.(Arg525His) | 25% | TP53 (c.472delC, p.Arg158fs*12), VAF 16%) | 46,XY,del(5q),del(7)(q22),add(10)(p?15) |
|  |  |  |  |  | c.1230+2T>C | p.? (Splicing Variant) | 33% | TP53 (c.320_321insA, p.(Tyr107fs*1), VAF 26%) |  |
| **Variants of Uncertain Significance (VUS)** | | | | | | | | | |
| PD8088a | No class | c.606C>G | p.(His202Gln) | 49% |  |  |  | NRAS (c.38G>A, p.(Gly12Asp), VAF 39%) | 46,XX |
|  |  |  |  |  |  |  |  | WT1 (c.1105C>T, p.(Arg369*), VAF 54%) |  |
|  |  |  |  |  |  |  |  | WT1 (c.792_793insC, p.(Val267fs*26), VAF 43%) |  |
| PD7988a | chromatin_ spliceosome | c.490C>T | p.(Arg164Trp) | 48% |  |  |  | DNMT3A (c.2645G>A, p.(Arg882His), VAF 42%) | 46,XX,i(7)(p10) |
|  |  |  |  |  |  |  |  | IDH2 (c.419G>A, p.(Arg140Gln), VAF 49%) |  |
|  |  |  |  |  |  |  |  | STAG2 (c.1810C>T, p.(Arg604*), VAF 6%) |  |
| PD10860a | chromatin_ spliceosome | c.1199G>A | p.(Arg400His) | 49% |  |  |  | RUNX1 (c.1110_1111insATGG, p.(Gly372fs*229), VAF 43%) | 46,XX,-12,+mar[10]/46,XX[5] |
| PD11213a | p53_ aneuploidy | c.465G>A | p.(Met155Ile) | 62% |  |  |  | TP53 (c.645T>G, p.(Ser215Arg), VAF 27%) | 45,XY,-5,add(5)(p11),del(6)(p11),add(7)(p15),-8,-17,add(17)(p11),-19,+mar1,+mar2,+mar3,inc |
|  |  |  |  |  |  |  |  | TP53 (c.560-1G>A, p.? (Splicing Variant), VAF 19%) |  |
| PD11223a | p53_ aneuploidy | c.124C>T | p.(Arg42Cys) | 48% |  |  |  | IDH1 (c.394C>G, p.(Arg132Gly), VAF 41%) | 45,XY,add(2)(p11),del(5)(q11),add(7)(q22orq31),add(15)(p11),-17[3]/46,XY[7] |
|  |  |  |  |  |  |  |  | TP53 (c.715A>G, p.(Asn239Asp), VAF 57%) |  |

**Suppl. Table 11: DDX41 mutations in the extension cohort.** Cases not classified as DDX41-mutated in the original publication are shown in **bold**. One variant, reclassified from pathogenic to a variant of uncertain significance (VUS), is shown in italic.

| **Pathogenic *DDX41* Variants** | | | | | | |
| --- | --- | --- | --- | --- | --- | --- |
| **Patient** | **germline *DDX41*** | | | **somatic *DDX41*** | | |
|  | **Codon** | **Protein** | **VAF** | **Codon** | **Protein** | **VAF** |
| 219-002 | c.1088_1090del | p.(Ser363del) | 48% | c.1574G>A | p.(Arg525His) | 19% |
| 234-002 | c.992_994del | p.(Lys331del) | 41% | c.1574G>A | p.(Arg525His) | 7% |
| **256-015** | **c.1088_1090del** | **p.(Ser363del)** | **46%** | **c.1574G>A** | **p.(Arg525His)** | **1%** |
| 230-009 | c.638C>T | p.(Pro213Leu) | 49% | c.1574G>A | p.(Arg525His) | 11% |
| **102-004** | **c.3G>A** | **p.(Met1Ile)** | **51%** | **c.545G>A** | **p.(Ser182Asn)** | **2%** |
| 104-002 | c.946_947del | p.(Met316Aspfs*31) | 45% | c.1589G>A | p.(Gly530Asp) | 16% |
| 154-002 | c.1298delC | p.(Pro433Argfs*28) | 45% | c.682G>T | p.(Gly228Cys) | 16% |
| 215-014 | c.1586_1587del | p.(Thr529Argfs*12) | 38% | c.1574G>A | p.(Arg525His) | 1% |
| 218-020 | c.1298dupC | p.(Pro434Alafs*25) | 45% | c.1574G>A | p.(Arg525His) | 1% |
| 219-003 | c.847delC | p.(Leu283Cysfs*21) | 46% | c.1574G>A | p.(Arg525His) | 9% |
| 219-008 | c.847delC | p.(Leu283Cysfs*21) | 44% | c.1589G>A | p.(Gly530Asp) | 24% |
| 220-001 | c.3G>A | p.(Met1Ile) | 47% | c.1574G>A | p.(Arg525His) | 7% |
| 230-006 | c.647dupT | p.(Ser217Ilefs*4) | 44% | c.1574G>A | p.(Arg525His) | 8% |
| 240-006 | c.305_306del | p.(Lys102Argfs*32) | 42% | c.1574G>A | p.(Arg525His) | 8% |
| 256-005 | c.415_418dup | p.(Arg140Glyfs*2) | 39% | c.1032C>G | p.(Asp344Glu) | 26% |
| 286-002 | c.1018dupT | p.(Tyr340Leufs*8) | 47% | c.1574G>A | p.(Arg525His) | 5% |
| **286-004** | **c.3G>A** | **p.(Met1Ile)** | **49%** | **c.1574G>A** | **p.(Arg525His)** | **1%** |
| 301-001 | c.475C>T | p.(Arg159*) | 50% | c.1574G>A | p.(Arg525His) | 27% |
| 302-005 | c.1105C>T | p.(Arg369*) | 49% | c.1574G>A | p.(Arg525His) | 17% |
| 312-020 | c.411delG | p.(Tyr138Metfs*16) | 49% | c.1574G>A | p.(Arg525His) | 17% |
| **331-005** | **c.1141A>T** | **p.(Lys381*)** | **52%** | **c.1574G>A** | **p.(Arg525His)** | **1%** |
| 346-001 | c.1230+2T>C | p.? (Splicing variant) | 48% | c.1589G>A | p.(Gly530Asp) | 4% |
| 346-011 | c.305_306del | p.(Lys102Argfs*32) | 43% | c.1574G>A | p.(Arg525His) | 10% |
| 402-008 | c.415_418dup | p.(Arg140Glyfs*2) | 38% | c.1574G>A | p.(Arg525His) | 1% |
| 350-002 | c.962C>T | p.(Pro321Leu) | 51% |  |  |  |
| 102-007 | c.430delA | p.(Thr144Profs*10) | 45% |  |  |  |
| **116-004** | **c.3G>A** | **p.(Met1Ile)** | **45%** |  |  |  |
| 213-005 | c.121C>T | p.(Gln41*) | 44% |  |  |  |
| 215-004 | c.1586_1587del | p.(Thr529Argfs*12) | 38% |  |  |  |
| 303-001 | c.415_418dup | p.(Arg140Glyfs*2) | 41% |  |  |  |
| 230-001 |  |  |  | c.1574G>A | p.(Arg525His) | 23% |
| 234-003 |  |  |  | c.622C>G | p.(Gln208Glu) | 12% |
| 256-004 |  |  |  | c.1019A>G | p.(Tyr340Cys) | 5% |
| 271-004 |  |  |  | c.1574G>A | p.(Arg525His) | 18% |
|  |  |  |  | c.475C>T | p.(Arg159*) | 17% |
| 280-006 |  |  |  | c.337G>A | p.(Glu113Lys) | 12% |
|  |  |  |  | c.645-1G>A | p.(?) Splicing variant | 12% |
| 101-012 |  |  |  | c.1574G>A | p.(Arg525His) | 9% |
|  |  |  |  | c.1768C>T | p.(His590Tyr) | 8% |
| 238-001 |  |  |  | c.1136C>T | p.(Pro379Leu) | 5% |
|  |  |  |  | c.1032C>A | p.(Asp344Glu) | 5% |
| **Variants of Uncertain Significance (VUS)** | | | | | | |
| 221-006 | c.907G>A | p.(Val303Met) | 47% |  |  |  |
| 235-002 | c.881G>T | p.(Cys294Phe) | 52% |  |  |  |
| *312-002* |  |  |  | *c.1720C>G* | *p.(Leu574Val)* | *20%* |

**Suppl. Table 12: Clinical characteristics of DDX41-mutated outlier cases identified by UMAP analysis across the primary and extension cohorts.** Abbr.: UMAP – Uniform Manifold Approximation and Projection; WBC – white blood cell count

| Cohort | Patient-ID | *DDX41-*Constellation | UMAP Cluster | Sex | Age (yrs.) | WBC (x10^9^/L) |
| --- | --- | --- | --- | --- | --- | --- |
| Primary | PD11215a | Somatic-Only  (2 somatic) | complex karyotype | male | 61 | 1.2 |
| Primary | PD8064a | Germline-Only | *FLT3*-ITD | female | 29 | 59.2 |
| Extension | 256-004 | Somatic-Only | complex karyotype | male | 75 | 3.9 |
| Extension | 213-005 | Germline-Only | mixed | male | 67 | 2.1 |
| Extension | 350-002 | Germline-Only | mixed | female | 77 | 16.5 |

3. List of Abbreviations

ACMG	American College of Medical Genetics and Genomics

AlloHSCT	Allogeneic hematopoietic stem-cell transplantation

AML	Acute myeloid leukemia

AMLSG	AML Study Group

ATRA	All-trans retinoic acid

CR	Complete remission

CR1	First complete remission

DBSCAN	Density-based spatial clustering of applications with noise

DDX41	DEAD-box helicase 41

EFS	Event-free survival

ECOG	Eastern Cooperative Oncology Group performance status

HMA	Hypomethylating Agent

HR	Hazard ratio

ICC	International Consensus Classification

ICE	Idarubicin/Cytarabine/Etoposide

ITD	Internal tandem duplication

LDAC	Low-Dose Cytarabine

MDS	Myelodysplastic syndrome

OS	Overall survival

RFS	Relapse-free survival

sAML	Secondary AML

tAML	Therapy-related AML

TKD	Tyrosine kinase domain

TP53	Tumor protein 53

UMAP	Uniform manifold approximation and projection

VAF	Variant allele frequency

VUS	Variant of uncertain significance

WBC	White blood cell count

WHO	World Health Organization
